# Effect of Phosphate-Solubilizing Bacteria on the Mobility of Insoluble Cadmium and Metabolic Analysis

**DOI:** 10.3390/ijerph15071330

**Published:** 2018-06-25

**Authors:** Ping Yang, Xue-Fang Zhou, Li-Li Wang, Qu-Sheng Li, Ting Zhou, Yu-Kun Chen, Zi-Yi Zhao, Bao-Yan He

**Affiliations:** School of Environment, Jinan University, Guangzhou 511443, China; yangp35@jnu.edu.cn (P.Y.); fangzhouujn@163.com (X.-F.Z.); wanglili@jnu.edu.cn (L.-L.W.); 1534281009@stu2015.jnu.edu.cn (T.Z.); 13424468075@163.com (Y.-K.C.); 15521384003@163.com (Z.-Y.Z.); thbyan@jnu.edu.cn (B.-Y.H.)

**Keywords:** phosphate-solubilizing bacteria, cadmium-mobilizing bacteria, metabolic pathway, organic acid

## Abstract

Phosphate-solubilizing bacteria (PSB) can promote plant growth by dissolving insoluble phosphate. Therefore, PSB may have the potential to improve the mobility of heavy metals in soils and enhance phytoextraction. This study isolated a few PSB strains that could dissolve CdCO_3_ and solid Cd in soil. Two typical PSB, namely, high- and low-Cd-mobilizing PSB (*Pseudomonas fluorescens* gim-3 and *Bacillus cereus* qh-35, respectively), were selected to analyze the metabolic profiles, metabolic pathways, and mechanisms of mobilization of insoluble Cd. A total of 34 metabolites secreted by the two PSB strains were identified. Gluconic acid was the main contributor to Cd dissolution (42.4%) in high-Cd-mobilizing PSB. By contrast, gluconic acid was not secreted in low-Cd-mobilizing PSB. Metabolic pathway analysis showed that gluconic acid was produced by the peripheral direct oxidation pathway. Hence, PSB with peripheral direct oxidation pathway were likely to have high-Cd-mobilizing capacity.

## 1. Introduction

Cadmium or cadmium compounds can increase the risk of lung cancer, kidney lesions, and bone damage [1,2,3,4]. Cadmium in soil can be assimilated by crops; because of which, it enters the human body through the food chain and adversely affects human health. Phytoextraction is an ecologically friendly and cost-effective technique that can be used to remove Cd from contaminated soils [5,6,7,8]. However, this process suffers from some limitations, such as the low mobility of the tightly bound fraction of Cd in soils with neutral pH, resulting in reduced uptake by plants [9].

To address such limitations, scientists have developed bacterium-assisted phytoextraction, in which bacteria are added to soil to facilitate Cd phytoextraction [10,11,12,13,14]. Bacteria such as *Bacillus pumilus* E2S2 increased *Sedum plumbizincicola* Cd uptake (43%) [15], and *Pseudomonas* sp. Lk9 increased Cd uptake (46.6%) of *Solanum nigrum* L. in multi-metal contaminated soil [16]. The added bacteria usually need to promote plant growth and increase the amount of metals absorbed by the tested plants [17,18]. Several studies have employed bacteria that were selected or tested for the presence of the enzyme 1-aminocyclopropane-1-carboxylate deaminase and the ability to synthesize indole-3-acetic acid and siderophores [19,20,21,22]. Nevertheless, few studies have investigated the potential role of an active phosphate solubilization system in bacteria in promoting heavy metal solubilization in soil. It has been reported that phosphate fertilizer can improve the phytoextraction efficiency of Cd by *Sedum* [23].

Phosphate-solubilizing bacteria (PSB) can improve plant growth in contaminated soils by supplying macronutrient phosphorus and are thus beneficial to Cd remediation [24]. PSB dissolves inorganic phosphates by secreting organic acids [25,26,27]. These organic acids enhance phosphate solubility by ionizing protons to decrease the pH and to combine PO_4_^3–^ to form HPO_4_^2–^ or H_2_PO_4_^–^. Organic acid anions can also form a complex with metal cations (Ca^2+^, Al^3+^, and Fe^3+^) and consequently, release PO_4_^3–^. In the same way, PSB-assisted phytoextraction may improve the mobility of Cd in soil, which has been reported by several studies [28,29]. For example, endophytic *Rahnella* sp. JN6 effectively solubilized 8.8 mg/L Cd and 133.54 mg/L phosphate in a culture solution and enhanced Cd accumulation in *Brassica napus* [30]. *Burkholderia* sp. J62 solubilized 25.8 mg/L Cd and 234 mg/L phosphate in a culture solution and increased the biomass of maize and tomato plants [31]. Decreases of the pH values in the culture solutions were considered their causes. However, the dissolving amount of cadmium was different at similar pH values in these studies. To our knowledge, organic acids of different types and quantities can dissolve varying amounts of phosphate and Cd [32,33]. So, we needed to identify the secreted organic acids to explain the differences in the insoluble Cd dissolved by different strains. Furthermore, the metabolic mechanisms involved in the production of these organic acids must be elucidated.

The present study aimed to (1) investigate the effects of PSB on CdCO_3_ solubility and Cd mobilization in soil collected from a Cd-polluted field; (2) research the differences between the secretions of high- and low-Cd-mobilizing PSB; and (3) explain the differences in the metabolic pathways of high- and low-Cd-mobilizing PSB.

## 2. Material and Methods 

### 2.1. PSB Isolation and Cd Solubility Tests

Soils were collected from a vegetable field located in a suburb of Guangzhou. The soil was polluted with Cd because of sewage irrigation 20 years ago. The soil properties were as follows: pH, 6.86 ± 0.11; total P, 2005.2 ± 58.8 mg/kg; available P, 94.0 ± 1.2 mg/kg; available N, 118.5 ± 5.3 mg/kg; organic C, 35.4 ± 1.2 g/kg; cation exchange capacity (CEC), 30.6 ± 1.1 cmol/kg; and total Cd, 1.952 ± 0.084 mg/kg. The speciation of Cd, as determined by the Tessier method [34], in the soil was as follows: exchangeable, 0.286 mg/kg; bound to carbonates, 0.239 mg/kg; bound to Fe–Mn oxides, 0.486 mg/kg; bound to organic matter, 0.189 mg/kg; and residual, 0.851 mg/kg.

Briefly, 10 g of soil was mixed with 90 mL of sterile water and shaken for 1 h to extract bacteria from the Cd-contaminated soil. Suitable dilutions were inoculated using a phosphate-solubilizing test agar medium containing (per liter) 10 g of glucose, 5 g of Ca_3_(PO_4_)_2_, 0.3 g of NaCl, 0.5343 g of MgSO_4_·7H_2_O, 0.0227 g of MnSO_4_·H_2_O, 0.3 g of KCl, 0.5 g of (NH_4_)_2_SO_4_, 0.03 g of FeSO_4_·7H_2_O, and 15 g of agar. The pH of the agar medium was adjusted to pH 7.0. After 7 days of plate incubation at 28 °C, colonies with clear halos considered to be PSB were counted. The predominant colonies were purified through re-streaking on a phosphate-solubilizing test agar plate.

Bacterial isolates were cultured in 50 mL of phosphate-solubilizing test liquid medium supplemented with 0.06 g/L CdCO_3_ (39.1 mg/L Cd), 0.668 g/L Ca_3_(PO_4_)_2_, and 1 g/L glucose (as nutrient-rich medium) or 0.06 g/L CdCO_3_ (39.1 mg/L Cd), Ca_3_(PO_4_)_2_ 0.334 g/L, glucose 1 g/L, and 10% concentration of the corresponding inorganic salts (as nutrient-poor medium). The inoculated media were incubated for 1, 3, 5, and 7 days on a rotary shaker at 28 °C, respectively. The mobility of the cadmium showed obvious differences between day 1 and day 7, so we took these two treatments to conduct follow-up tests. A separate liquid medium inoculated with sterile water served as the control treatment. Three replicates were used for each experiment. After the culture solution was centrifuged at 1800× *g* for 10 min, the supernatant was passed through a 0.22 μm filter. A part of each filtrate was used as sample for gas chromatography–mass spectrometry (GC–MS) determination, and the other part was used for measuring pH and solubilized P and Cd concentrations. The pH of each filtrate was measured using a pH meter. Solubilized P concentration was measured by colorimetric Mo-blue method, using UV mini-1240 UV–Vis spectrophotometer (Shimadzu, Kyoto, Japan). The concentration of soluble Cd was determined by an atomic absorption spectrometer in flame mode (AAS, PerkinElmer, Waltham, MA, USA). A total of 10 bacterial strains that can simultaneously dissolve phosphate and cadmium carbonate were screened (data not shown). The PSB with the highest (strain gim-3) and lowest (strain qh-35) dissolved cadmium concentrations were selected for 16S rDNA gene sequencing, which was conducted by the BGI GROUP, Guangzhou, China. The 16S rDNA sequence showed that strain qh-35 was very closely related to *B. cereus* and strain gim-3 was very closely related to *P. fluorescens* (Text S1 and Text S2,)*.* Strain qh-35 was gram-positive. Its colonies were small, suborbicular, and the colony surface was smooth and transparent (Figure S1). Strain gim-3 was gram-negative. Its colonies were small, round, and opaque with a white spot. The colony surface was moist and flat (Figure S2). Subsequently, physiological, and biochemical tests were conducted using an API 50 CHB, API 20 E, and API 20 NE system (Biomerieux, Marcy-l’Étoile, France) (Tables S1 and S2).

### 2.2. Effects of PSB on Cd Mobility in Contaminated Soil

Pure bacterial isolates (*B. cereus* or *P. fluorescens*) were cultivated in 100 mL of nutrient-rich medium containing 1 g of sterile Cd-polluted soil on a rotary shaker at 28 °C. A control sample was prepared by adding the same amount of sterile water instead of bacterial inoculate. After 1 or 7 days of incubation, the soil suspensions were centrifuged at 1800× *g* for 10 min and filtered. The Cd concentration in the filtrate was measured by atomic absorption spectroscopy (AAS).

### 2.3. Determination of PSB Secretions and Organic Acids

The experimental process referred to some literature [35,36]. Each culture filtrate sample (0.5 mL) of the CdCO_3_ solubility tests was added to test tubes and freeze dried. The dried residue was derivatized by 20 μL of methoxyamine hydrochloride (20 mg/mL in pyridine at 37 °C for 2 h) and 70 μL *N*-methyl-*N*-(trimethylsilyl) trifluoroacetamide (at 37 °C for 30 min). Then, n-hexane (410 μL) was added to form a total volume of 0.5 mL.

Samples (1 μL) were injected into a gas chromatography (GC) (GC-2010 Plus, Shimadzu, Kyoto, Japan) in splitless mode by an autosampler (AOC-20s, Shimadzu, Kyoto, Japan) and autoinjector (AOC-20i, Shimadzu, Kyoto, Japan). GC analysis was carried out on a Rxi-5MS with a capillary column (30 m × 0.25 mm × 0.25 μm, Thermo Fisher, Waltham, MA, USA). The injection, interface, and ion-source temperatures were adjusted to 230 °C, 250 °C, and 220 °C, respectively. The helium flow rate was 1 mL/min. The column temperature was controlled as follows: maintained for 1 min at 70 °C, ramped to 76 °C in 6 min, then ramped to 300 °C in 44.8 min, and held for 10 min at 300 °C. The column end was introduced into a quadrupole electron impact ionization mass spectrometer (GCMS-QP2010 Ultra, Shimadzu, Kyoto, Japan). Mass spectra were recorded at 2 scans/s under *m*/*z* 50–600 scanning range.

The raw GC–MS chromatogram was automatically analyzed using the automatic mass spectral deconvolution and identification system (AMDIS) and compared with the NIST05 database [37,38]. If the similarity was greater than 80% and the retention index difference was less than 20, then the compounds were identified [39,40]. The retention index was obtained by measuring the retention time of n-alkanes (0.5 μL, n-alkane C8–C40, SUPELCO, Bellefonte, PA, USA) in the blank solution. 

All secreted organic acids were quantified by the method of internal standard method [41,42]. The peak area was extracted and calculated using metabolomics ion-based data extraction algorithm (MET-IDEA), which selected the first or second strong peak as the mark peak (Table S3). 

### 2.4. The Ability of PSB-Secreted Organic Acids to Solubilize Cd

To determine the Cd-solubilizing ability of the detected organic acids, 10 mg of CdCO_3_ and 20 mL of a series of organic acid solutions with different concentrations were added into a 50 mL tube. The mixture was shaken for 1 day at room temperature. A control was prepared by adding the same amount of sterile water instead of organic acid solution. Three replicates were used for each experiment. After the solution was centrifuged at 1800× *g* for 10 min and filtered, the Cd concentration in the filtrate was measured by AAS. A relationship between Cd concentration and organic acid concentration was established. A curve showing the cadmium-solubilizing ability (CdCO_3_) of each organic acid tested is presented in Figure S3. The Cd concentration dissolved by organic acids secreted from bacteria can be calculated based on this curve.

### 2.5. Statistical Analysis

Statistical analysis was conducted using SPSS (IBM, Armonk, NY, USA) 19. Data were tested at significance levels of *p* < 0.05 by independent-sample Mann–Whitney U test (for non-parametric test), student’s *t*-test (for two samples), and ANOVA Duncan test (for three or more samples).

## 3. Results and Discussion 

### 3.1. Effects of PSB on Solubilization of Cd Carbonate in Liquid Medium

Table 1 shows the effects of *Bacillus cereus* and *Pseudomonas fluorescens* on the solubility of cadmium and phosphate. Both of them not only dissolved a considerable amount of phosphate but also solubilized Cd carbonate. After the strains were incubated in the liquid medium for 1 and 7 days, the fraction of dissolved Cd increased from 9.4% to 92%. In addition, *P. fluorescens* was superior to *B. cereus* in solubilizing Cd. Therefore, we designated *P. fluorescens* and *B. cereus* as high- and low-Cd-mobilizing PSB, respectively.

*B. cereus* grew well in the nutrient-rich medium. The dissolving amount of phosphate and Cd increased with the increase of incubation time. The amount of dissolved Cd was 16.9 mg/L (43.3%) on the first day and increased to 20.0 mg/L (51.2%) after 7 days. The pH of the solution decreased from 6.24 to 5.38. By contrast, the growth and Cd-dissolving ability of the strain were restrained in the nutrient-poor medium because of insufficient nutrients. The levels of dissolved Cd were 4.4 mg/L (11.3%) and 5.34 mg/L (13.7%) on days 1 and 7, respectively, and the pH did not change.

*P. fluorescens* also grew well in the nutrient-rich medium. But the growth rate of *P. fluorescens* was faster than that of *B. cereus*. So, the amounts of dissolved phosphate and Cd (23.0 mg/L and 76.6%) reached high values on day 1. After 7 days of incubation, the bacteria died, disintegrated, and adsorbed dissolved Cd, thereby decreasing the Cd concentration to 12.0 mg/L (30.7%). The pH increased from 5.00 to 6.36. In the nutrient-poor medium, bacterial growth was delayed because of insufficient nutrients. The amount of dissolved Cd was 5.18 mg/L (13.3%) on the first day and increased to 36.0 mg/L (92.3%) after 7 days. The pH decreased from 6.28 to 4.09.

PSB dissolved inorganic phosphate mainly by acidification. Thus, PSB can facilitate the mobility and bioavailability of heavy metals in soils. Similarly, the addition of *Pseudomonas* sp. TLC 6–6.5–4 to the Bushnell Hass (BH) medium supplemented with CuCO_3_ solubilized 41.8 mg/L Cu and decreased the pH value from 7 to 4.9 [43]. Previous studies had documented that acidification of bacteria had a good effect on the solubility and phytoextraction of Cd [30,31]. Our results also showed that the concentrations of dissolved phosphate and Cd were inversely related to the pH value. With the pH value from 6.49 to 4.09 in different media of two strains, the amount of cadmium dissolved from 4.40 mg/L to 36.0 mg/L.

### 3.2. Effects of Cd-Mobilizing PSB on Mobility of Soil Cd

Table 2 showed the effects of PSB on Cd mobility in Cd-polluted soil. In *P. fluorescens*, after the control data were deducted, 0.18 and 0.39 mg/kg Cd were dissolved in the 1- and 7-day culture. These amounts accounted for 9.2% and 20.0% of the total Cd in the soil, accounting for 34.3% and 74.3% of the sum of the exchangeable and carbonate-bound Cd. In *B. cereus*, only 0.04 mg/kg Cd was dissolved after being cultured for 1 day compared with the non-inoculated control. This amount was 2.0% of the total Cd and 7.6% of the sum of the exchangeable and carbonate-bound Cd. The amount of dissolved Cd in *B. cereus* for 7 days was close to that of the control group.

Ma et al. reported that *Psychrobacter* sp. SRS8 and *B. cereus* SRA10 mobilized 16 and 12 mg/kg Ni in soil [44,45]. Rajkumar et al. observed that *Pseudomonas* sp. PsM6 and *Bacillus weihenstephanesis* SM3 mobilized 14 and 8 mg/kg Zn in soil, respectively [46,47]. In the present study, high-Cd-mobilizing PSB *P. fluorescens* promoted the release of 0.39 mg/kg Cd from the Cd-polluted soil, which could have the potential to enhance the extraction of Cd by plants. He et al. reported that *Rahnella* sp. JN6 increased 0.46 mg/kg Cd in *B. napus* rhizosphere soil, which doubled the content of Cd in the plant [30]. Jiang et al. reported that *Burkholderia* sp. J62 increased 0.50 mg/kg Cd in maize rhizosphere soil and 0.88 mg/kg Cd in tomato rhizosphere soil, which greatly increased the uptake of Cd in maize root and tomato shoot [31].

### 3.3. Metabolite Analysis and Their Cd-Solubilizing Abilities

*P. fluorescens* and *B. cereus* grew well in the nutrient-rich liquid medium with CdCO_3_ on culture day 1. Therefore, we selected this culture solution for further secretions analysis. A total of 34 secretions were identified by GC–MS (Table S3). Mann–Whitney U test analysis showed that there were significant (*p* < 0.05) differences in the secretions of *B. cereus* and *P. fluorescens* except glycerol and ribose. In *B. cereus*, the majority of secretions were monosaccharides, followed by organic acid. In *P. fluorescens*, the major secretions were organic acids.

Table 3 showed the concentrations of organic acids and their Cd-solubilizing abilities. The secretion of *P. fluorescens* was mostly composed of gluconic acid, which can dissolve 13.1 mg/L Cd, followed by oxalic acid. On the other hand, the secretion of *B. cereus* was mostly comprised of pyruvic acid, which can dissolve 4.52 mg/L Cd, followed by glycolic acid. The total concentrations of Cd solubilized by all the secreted organic acids in *B. cereus* and *P. fluorescens* equaled 19.2 and 30.8 mg/L, respectively, which were similar to Cd concentrations (16.94 and 29.96 mg/L, respectively) in the nutrient-rich medium after 1 day of inoculation (Table 1). This indicated that the calculated results of the Cd-solubilizing abilities of the secreted organic acids were consistent with the actual detected results. The high solubilizing effect of *P. fluorescens* for Cd mainly resulted from large amounts of gluconic acid (75.9% of the total organic acid concentration) and oxalic acid (7.0% of the total organic acid concentration) produced (Table 3). The amount of Cd solubilized by gluconic acid and oxalic acid reached 42.4% and 24.4%, respectively, of the total concentration of Cd solubilized by *P. fluorescens*. This was consistent with previous findings that the *Pseudomonas* species release P from sparingly soluble mineral phosphates by producing high levels of gluconic acid from extracellular glucose [48,49].

### 3.4. Metabolic Pathway Analysis

**Figure 1:**
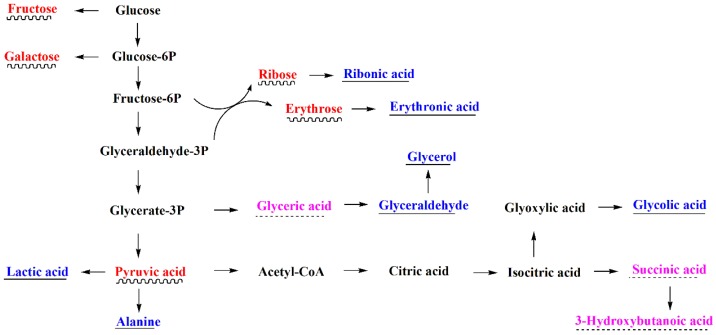
The primary metabolism showing the major compounds that were detected in *B. cereus* qh-35. The red-labeled and wavy-line-underscored metabolites are those in large quantities. The blue-labeled and straight-line-underscored metabolites are those in moderate quantities. The purple-labeled and dotted-line-underscored metabolites are those in small quantities.

**Figure 2:**
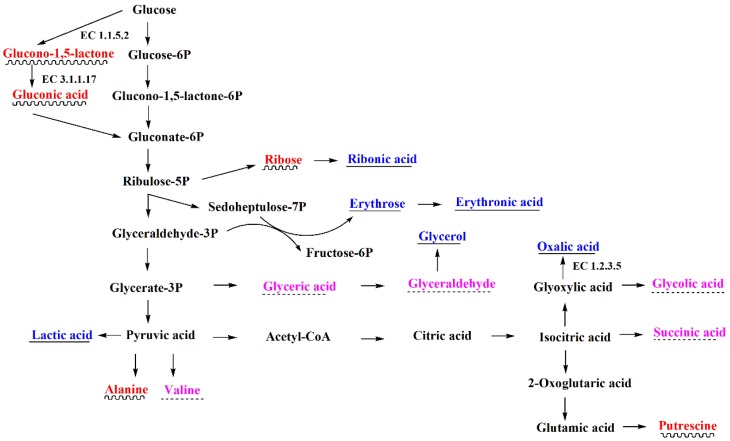
The primary metabolism showing the major compounds that were detected in *P. fluorescens* gim-3. The red-labeled and wavy-line-underscored metabolites are those in large quantities. The blue-labeled and straight-line-underscored metabolites are those in moderate quantities. The purple-labeled and dotted-line-underscored metabolites are those in small quantities.

Based on the previous literature [49] and the Kyoto Encyclopedia of Genes and Genomes (KEGG) database [50], lactic acid and pyruvic acid were mainly produced by glycolysis. Succinic acid was produced by TCA cycle. Gluconic acid was produced through the periplasmic direct oxidation pathway and the pentose phosphate pathway. Glycolic acid and oxalic acid were produced through the glyoxylate and dicarboxylate metabolism. Therefore, the major metabolites secreted by the two strains were mainly produced by carbon metabolism, and their primary metabolic networks compiled from KEGG were presented in Figure 1 and Figure 2, respectively.

Figure 1 and Figure 2 showed that glucose catabolism was considerably different between *B. cereus* and *P. fluorescens*. *B. cereus* produced energy and intermediates for biosynthesis through glycolysis, which was similar to *Bacillus* sp. [49]. A large amount of pyruvic acid was produced by glycolysis. However, *P. fluorescens* converted extracellular glucose to gluconic acid, as in previous studies [51,52]. Generally, two glucose 1-dehydrogenases (EC 1.1.5.2 and EC 1.1.1.47) can convert glucose into gluconolactone, which was further hydrolyzed by gluconolactonase (EC 3.1.1.17) into gluconic acid. EC 1.1.5.2 occurred in the periplasm and was controlled by the *gcd* gene [53,54,55,56], while EC 1.1.1.47 occurred in the intracellular and was controlled by the *gdh* gene. They were involved in the periplasmic direct oxidation pathway and the pentose phosphate pathway, respectively. In P-limiting conditions, the direct oxidation pathway predominated in the *Pseudomonas* species [48]. In present study, glucose was not detected in the culture medium of *P. fluorescens* gim-3, but a large amount of gluconic acid was detected (Table S3). This indicated that the conversion of glucose was very fast. This can only be achieved by extracellular oxidation. Therefore, in *P. fluorescens* gim-3, glucose catabolism primarily occurred through the periplasmic direct oxidation pathway. The isolated strain of *P. fluorescens* gim-3 could produce EC 1.1.5.2 and EC 3.1.1.17, but *B. cereus* qh-35 could not produce them.

Furthermore, the products of glyoxylate and dicarboxylate metabolism of *B. cereus* and *P. fluorescens* were different (Figure 1 and Figure 2). *P. fluorescens* can produce oxalic acid and glycolic acid. However, *B. cereus* only produced glycolic acid. Therefore, the strain of *P. fluorescens* gim-3 isolated in this study might have glyoxylate oxidase (EC 1.2.3.5).

## 4. Conclusions

In this study, two PSB strains were isolated from the soil. Their metabolism and abilities to solubilize carbonate Cd and soil Cd were investigated. *P. fluorescens* gim-3, a high-Cd-mobilizing PSB, secreted large amounts of gluconic acid and dissolved more Cd. *B. cereus* qh-35, a low-Cd-mobilizing PSB, did not secrete gluconic acid and dissolved less Cd. Metabolic pathway analysis in bacteria showed that gluconic acid in *P. fluorescens* gim-3 was produced by the peripheral direct oxidation pathway, which was controlled by EC 1.1.5.2 and EC 3.1.1.17. Therefore, PSB with the peripheral direct oxidation pathway were likely to have high-Cd-mobilizing ability. PSB, by increasing cadmium mobility, may increase metal toxicity to flora and fauna, for example, on various invertebrates that have skin in contact with moist soil (such as eisenia) or even vertebrate eggs with semi-permeable shell (such as lizards). Further research will be conducted on this issue in the future.

## Figures and Tables

**Table 1:** Effect of *B. cereus* and *P. fluorescens* on the solubility of cadmium (CdCO_3_) and phosphate (Ca_3_(PO_4_)_2_).

Treatment	1 Day				7 Days			
	PO_4_^3–^ (mg/L)	Cd^2+^ (mg/L)	Cd^2+^ Dissolution Rate (%)	pH	PO_4_^3–^ (mg/L)	Cd^2+^ (mg/L)	Cd^2+^ Dissolution Rate (%)	pH
*B. cereus* ^1^	6.16 ± 0.17 ^bA^	16.94 ± 0.80 ^bA^	43.32	6.24 ± 0.05 ^bB^	10.06 ± 0.90 ^bB^	20.03 ± 1.42 ^dB^	51.23	5.38 ± 0.24 ^bA^
*P. fluorescens* ^1^	31.82 ± 2.13 ^cA^	29.96 ± 2.84 ^cB^	76.62	5.00 ± 0.11 ^aA^	30.55 ± 1.34 ^cA^	12.00 ± 0.77 ^cA^	30.69	6.36 ± 0.05 ^cB^
*B. cereus* ^2^	4.31 ± 0.19 ^abA^	4.40 ± 0.15 ^aA^	11.26	6.49 ± 0.03 ^cA^	4.04 ± 0.20 ^aA^	5.34 ± 0.11 ^bB^	13.66	6.43 ± 0.06 ^cA^
*P. fluorescens* ^2^	4.66 ± 0.23 ^abA^	5.18 ± 0.04 ^aA^	13.26	6.28 ± 0.03 ^bB^	64.27 ± 2.45 ^dB^	35.98 ± 0.56 ^eB^	92.03	4.09 ± 0.33 ^aA^
control	3.69 ± 0.30 ^aA^	2.87 ± 0.24 ^aA^	7.34	6.71 ± 0.04 ^dB^	3.76 ± 0.32 ^aA^	3.68 ± 0.35 ^aB^	9.41	6.54 ± 0.04 ^cA^

The superscript ^1^ indicates 100% liquid phosphate-solubilizing functional medium (pH 7.0–7.5) (called nutrient-rich medium) containing 0.3 g/L NaCl, 0.5343 g/L MgSO_4_·7H_2_O, 0.0227 g/L MnSO_4_·H_2_O, 0.3 g/L KCl, 0.5 g/L (NH_4_)_2_SO_4_, 0.03 g/L FeSO_4_·7H_2_O, 1 g/L glucose, 0.668 g/L Ca_3_(PO_4_)_2_, and 0.06 g/L CdCO_3_ (39.1 mg/L Cd). The superscript ^2^ was 10% liquid phosphate-solubilizing functional medium (pH 7.0–7.5) (called nutrient-poor medium) containing 0.03 g/L NaCl, 0.05343 g/L MgSO_4_·7H_2_O, 0.00227 g/L MnSO_4_·H_2_O, 0.03 g/L KCl, 0.05 g/L (NH_4_)_2_SO_4_, 0.003 g/L FeSO_4_·7H_2_O, 1 g/L glucose, 0.334 g/L Ca_3_(PO_4_)_2_, and 0.06 g/L CdCO_3_ (39.1 mg/L Cd). Data include mean ± standard deviation (*SD*) of three replicates (*n* = 3). Different small letters in each column indicate significant differences at the *p* < 0.05 level in accordance with the ANOVA Duncan test. Different capital letters in each row indicate that the pH and PO_4_^3–^ and Cd^2+^ concentrations at days 1 and 7 were significantly different at *p* < 0.05 in accordance with student’s *t* test.

**Table 2:** Effect of *B. cereus* and *P. fluorescens* on cadmium mobility in Cd-polluted soil.

Treatment	1 Day					7 Days				
	Dissolved Cd^2+^ (mg/kg Dry Soil)	pH	Net Dissolved Cd^2+^ (mg/kg Dry Soil)	Ratio of Net Dissolved Cd^2+^ to Total Cadmium in Soil	Ratio of Net Dissolved Cd^2+^ to the Exchangeable and Carbonate-Bound Cadmium	Dissolved Cd^2+^ (mg/kg Dry Soil)	pH	Net Dissolved Cd^2+^ (mg/kg Dry Soil)	Ratio of Net Dissolved Cd^2+^ to Total Cadmium in Soil	Ratio of Net Dissolved Cd^2+^ to the Exchangeable and Carbonate-Bound Cadmium
B. cereus	0.08 ± 0.03 ^a^	6.55 ± 0.07 ^b^	0.04	2.0%	7.6%	0.03 ± 0.01 ^a^	6.66 ± 0.09 ^b^	-	-	-
P. fluorescens	0.22 ± 0.03 ^b^	4.42 ± 0.05 ^a^	0.18	9.2%	34.3%	0.43 ± 0.08 ^b^	4.46 ± 0.18 ^a^	0.39	20.0%	74.3%
control	0.04 ± 0.02 ^a^	6.57 ± 0.20 ^b^				0.04 ± 0.02 ^a^	6.48 ± 0.20 ^b^			

Data are expressed as mean ± SD of three replicates (*n* = 3). Different small letters in each column indicate significant differences at *p* < 0.05 in the ANOVA Duncan test. The net dissolved Cd is equal to the dissolved Cd in the experimental group minus the dissolved Cd in the control group.

**Table 3:** Concentration and Cd-solubilizing ability of organic acids.

Organic acid	Concentration in *B. cereus* (mg/L)	Cd-Solubilizing Ability in *B. cereus* (mg/L)	Concentration in *P. fluorescens* (mg/L)	Cd-Solubilizing Ability in *P. fluorescens* (mg/L)
Pyruvic acid	9.738	4.52	n. d.	n. d.
Lactic acid	1.988	2.11	1.193	1.80
Glycolic acid	5.382	2.95	0.339	-
Oxalic acid	n. d.	n. d.	6.962	7.52
3-Hydroxy butyric acid	0.783	-	n. d.	n. d.
Succinic acid	1.098	2.28	0.854	-
Glyceric acid	0.998	-	0.431	-
Gluconic acid	n. d.	n. d.	75.315	13.08
Erythronic acid	2.120	1.51	5.260	2.22
Ribonic acid	3.242	1.77	3.554	1.84
3-Hydroxy propionic acid	0.087	-	n. d.	n. d.
2,4-Dihydroxy butanoic acid	1.346	1.34	2.203	1.53
3,4-Dihydroxy butanoic acid	1.255	1.32	2.084	1.57
Hexadecanoic acid	1.466	1.38	1.079	1.26

The “-” means the concentration of organic acid was not suitable for calculating the Cd-solubilizing ability, because its value was lower than the minimum value (1 mg/L) of the soluble cadmium capacity curve. The Cd-solubilizing ability of the organic acids refers to the amount of cadmium (CdCO_3_) dissolved under the corresponding organic acid concentration (Figure S3).

## Supplementary Materials

The following are available online at http://www.mdpi.com/1660-4601/15/7/1330/s1, Figure S1: Colony morphology of Bacillus cereus qh-35, Figure S2: Colony morphology of *Pseudomonas fluorescens* gim-3, Figure S3: Curve of the cadmium (CdCO_3_) solubilization ability of organic acids, Table S1: The physiological and biochemical reactions of *Bacillus cereus* qh-35, Table S2: The physiological and biochemical reactions of *Pseudomonas fluorescens* gim-3, Table S3: Secretions of *B. cereus* and *P. fluorescens*, Text S1: The 16S rDNA gene sequences of Bacillus cereus qh-35, Text S2: The 16S rDNA gene sequences of *Pseudomonas fluorescens* gim-3.
