# Supplementary material for: Effect of Phosphate-Solubilizing Bacteria on the Mobility of Insoluble Cadmium and Metabolic Analysis
Source: Int J Environ Res Public Health. 2018 Jun 25;15(7):1330. doi: 10.3390/ijerph15071330 (PMC6068833; doi:10.3390/ijerph15071330)
Supplement: Supplementary file 1 [file ijerph-15-01330-s001.pdf]

Appendix A. Supplementary data

Effect of phosphate-solubilizing bacteria on the mobility of insoluble cadmium and metabolic analysis

Ping Yang, Xue-Fang Zhou, Li-Li Wang, Qu-Sheng Li*, Ting Zhou, Yu-Kun Chen, Zi-Yi Zhao, Bao-Yan He

Corresponding Author: Qu-Sheng Li, School of Environment, Jinan University, Guangzhou, China, E-mail: liqusheng@21cn.com

The supplementary material includes Figure S1, Figure S2, FigureS3, Table S1, Table S2, Table S3, Text S1, and Text S2.

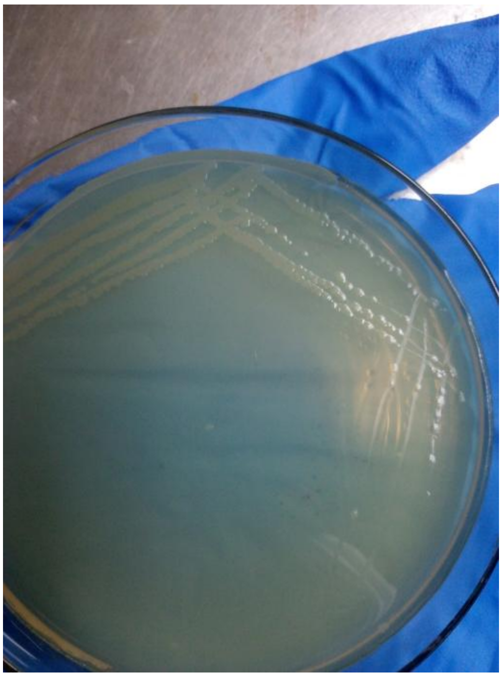

Figure S1. Colony morphology of *Bacillus cereus* qh-35**.**

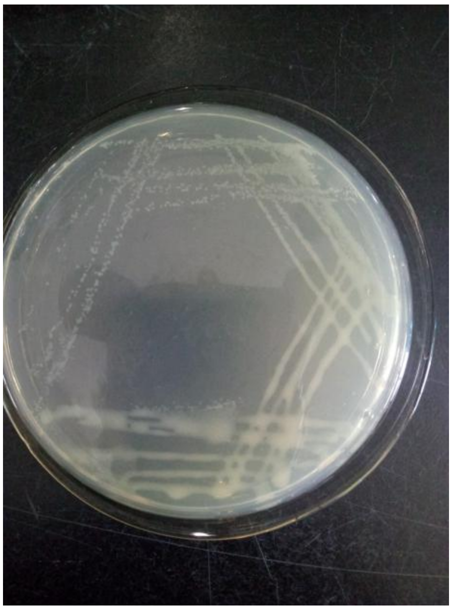

Figure S2. Colony morphology of *Pseudomonas fluorescens* gim-3**.**

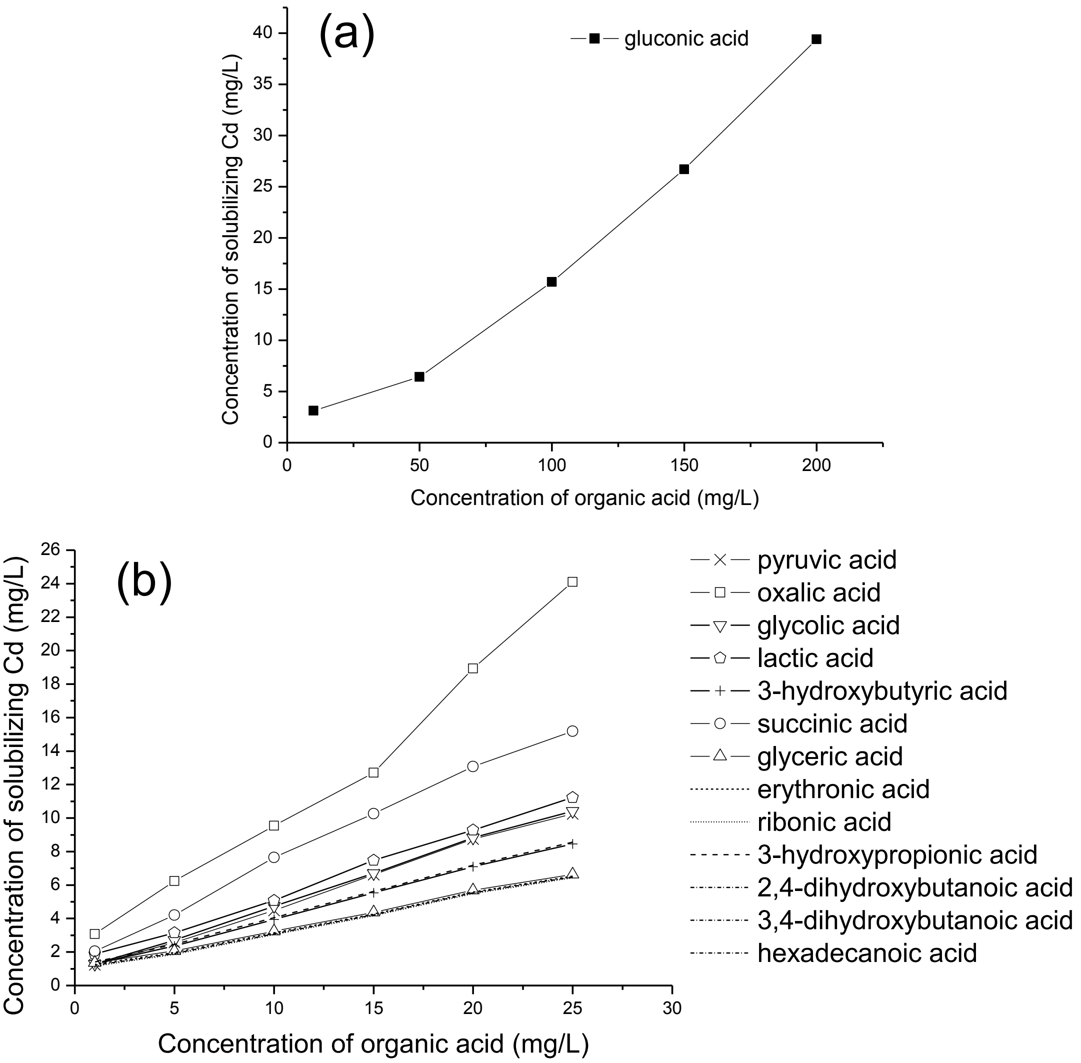

Figure S3. Curve of the cadmium ($CdCO_3$) solubilization ability of organic acids.

Dicarboxylic acid exhibits higher efficiency in dissolving Cd than monocarboxylic acids, although succinic acid has high pK_a (pK_{a1} = 4.21). The acid dissolution and coordination reactions of organic acids can stimulate the release of non-soluble compounds. Acid dissolution depends on pK_a value and concentrations of organic acids. The coordination reaction depends on the structure of organic acids. Obviously the first carboxyl-dissociated and -coordinated Cd greatly promote the dissociation and coordination of the second carboxyl. Although pyruvic acid has a small pK_a (pK_a = 2.49), the solubilizing Cd effect is similar to that of alpha-hydroxyl carboxylic acids, because the carbonyl group is much weaker than hydroxyl coordination. Gluconic acid (pK_a = 3.86), oxalic acid (pK_{a1} = 1.22), glycolic acid (pK_a = 3.83), lactic acid (pK_a = 3.86), 3-hydroxybutyric acid (pK_a = 4.36), glyceric acid (pK_a = 3.42), erythronic acid (pK_a = 3.47), ribonic acid (pK_a = 3.38), 3-hydroxypropionic acid (pK_a = 4.39), 2,4-dihydroxybutanoic acid (pK_a = 3.71), 3,4-dihydroxybutanoic acid (pK_a = 4.27), and hexadecanoic acid (pK_a = 4.78).

Table S1. The physiological and biochemical reactions of *Bacillus cereus* qh-35.

API 50 CHB	Strain qh-35
Assimilation of	
Glycerol (GLY)	−
Erythritol (ERY)	−
D-Arabinose (DARA)	−
L-Arabinose (LARA)	−
Ribose (RIB)	+
D-Xylose (DXYL)	−
L-Xylose (LXYL)	−
Adonitol (ADO)	−
Methyl-β-D-xylopyranoside (MDX)	−
Galactose (GAL)	−
Glucose (GLU)	+
Fructose (FRU)	+
Mannose (MNE)	−
Sorbose (SBE)	−
Rhamnose (RHA)	−
Dulcitol (DUL)	−
Inositol (INO)	−
Mannitol (MAN)	−
Sorbitol (SOB)	−
Methyl-α-D-mannopyranoside (MDM)	−
Methyl-α-D-glucopyranoside (MDG)	−
N-acetyl-glucosamine (NAG)	+
Amygdalin (AMY)	−
Arbutin (ARB)	−
Esculin (ESC)	+
Salicin (SAL)	−
Cellobiose (CEL)	−
Maltose (MAL)	+
Lactose (LAC)	−
Melibiose (MEL)	−
Saccharose (SAC)	−
Trehalose (TRE)	+
Inulin (INU)	−
Melezitose (MLZ)	−
Raffinose (RAF)	−
Acid modified starch (AMD)	+
Glycogen (GLYG)	+
Xylitol (XLT)	−
Gentiobiose (GEN)	−
Turanose (TUR)	−
Lyxose (LYX)	−
Tagatose (TAG)	−
D-Fucose (DFUC)	−
L-Fucose (LFUC)	−
D-Arabinitol (DARL)	−
L-Arabinitol (LARL)	−
Potassium gluconate (GNT)	−
Potassium 2-ketogluconate (2KG)	−

<table>
<tr><td>Potassium 5-ketogluconate (5KG)</td><td>−</td></tr>
<tr><td>API 20 E</td><td>Strain qh-35</td></tr>
<tr><td>o-Nitrophenyl-β-D-galactosidase (ONPG)</td><td>−</td></tr>
<tr><td>Arginine dihydrolase (ADH)</td><td>−</td></tr>
<tr><td>Lysine decarboxylase (LDC)</td><td>−</td></tr>
<tr><td>Ornithine decarboxylase (ODC)</td><td>−</td></tr>
<tr><td>Assimilation of citrate (|CIT|)</td><td>−</td></tr>
<tr><td>H_2S production (H_2S)</td><td>−</td></tr>
<tr><td>Urease (URE)</td><td>+</td></tr>
<tr><td>Tryptophan deaminase (TDA)</td><td>−</td></tr>
<tr><td>Indole production (IND)</td><td>−</td></tr>
<tr><td>Voges-Proskauer test (|VP|)</td><td>+</td></tr>
<tr><td>Gelatin hydrolysis (|GEL|) (protease)</td><td>+</td></tr>
<tr><td>Glucose fermentation or oxidation (GLU)</td><td>−</td></tr>
<tr><td>Identification</td><td>Bacillus cereus</td></tr>
</table>

Table S2. The physiological and biochemical reactions of *Pseudomonas fluorescens* gim-3.

API 20 NE	Strain gim-3
Nitrate reduction (NO3)	+
Tryptophan producing indole (TRP)	−
Glucose fermentation (GLU)	−
Arginine dihydrolase (ADH)	+
Urease (URE)	−
Esculine hydrolysis (ESC) (β-glucosidase)	−
Gelatin hydrolysis (GEL) (protease)	+
p-Nitrophenyl-β-D-galactosidase (PNPG)	−
Assimilation of	
Glucose (GLU)	+
Arabinose (ARA)	+
Mannose (MNE)	+
Mannitol (MAN)	+
N-acetyl-glucosamine (NAG)	+
Maltose (MAL)	−
Potassium gluconate (GNT)	+
Capric acid (CAP)	−
Adipic acid (ADI)	−
Malic acid (MLT)	+
Citrate (CIT)	+
Phenylacetic acid (PAC)	−
Oxidase (OX)	+
Identification	*Pseudomonas fluorescens*

Table S3. Secretions of *B. cereus* and *P. fluorescens.*

Time	Compound	Formula	RI calculation	RI reference	Derivatization	Mark peak	Peak area ($\times 10^5$) [a]	Peak area ($\times 10^5$) [b]
6.476	1-Hexanol	C6H14O	991.1	982	O-TMS	159	89.62626	84.26175
8.732	Pyruvic acid [a]	C3H4O3	1050.2	-	MO, O-TMS	73	3.12031	-
9.116	Lactic acid	C3H6O3	1060.2	1051	O,O-TMS	73	3.96691	1.76053
9.739	Glycolic acid	C2H4O3	1076.5	1078	O,O-TMS	73	11.16354	0.51969
10.708	Alanine	C3H7O2N	1102.1	1093	N,O-TMS	116	1.20397	17.27514
11.008	Hydroxylamine [b]	H3ON	1110.6	1123	N,N,O-TMS	73	-	0.32077
12.046	Oxalic acid [b]	C2H2O4	1140.3	1131	O,O-TMS	73	-	1.37809
12.238	3-Hydroxypropionic acid [a]	C3H6O3	1145.7	1144	O,O-TMS	177	0.07348	-
12.783	3-Hydroxybutyric acid [a]	C4H8O3	1161.3	1163	O,O-TMS	73	0.55035	-
14.569	Valine [b]	C5H11O2N	1213.8	1221	N,O-TMS	144	-	0.30108
14.806	Glyceraldehyde	C3H6O3	1221.4	-	MO, O,O-TMS	73	1.88081	0.60568
16.143	Ethanolamine	C2H7ON	1264.3	1281	N,N,O-TMS	174	0.21725	0.55529
16.309	Phosphoric acid	H3O4P	1269.5	1283	O,O,O-TMS	299	9.72631	22.67024
16.494	Glycerol	C3H8O3	1275.5	1282	O,O,O-TMS	73	2.81253	2.62104
17.614	Succinic acid	C4H6O4	1312.6	1314	O,O-TMS	73	0.85247	0.49004
18.093	Glyceric acid	C3H6O4	1329.5	1336	O,O,O-TMS	73	1.73067	0.55222
20.369	2,4-Dihydroxybutanoic acid	C4H8O4	1410.9	1431	O,O,O-TMS	103	0.64563	0.78120
20.934	3,4-Dihydroxybutanoic acid	C4H8O4	1432.7	1448	O,O,O-TMS	73	0.60173	0.73906
21.101	Erythrose	C4H8O4	1439.1	-	MO, O,O,O-TMS	73	2.73090	1.76661
21.483	Erythrose	C4H8O4	1453.7	-	MO, O,O,O-TMS	73	13.75517	8.82034
22.773	Salicylic acid	C7H6O3	1503.4	1507	O,O-T	73	0.95474	0.7060

	(internal reference)				MS			8
23.705	Erythronic acid	C4H8O5	1541.8	-	O,O,O, O-TMS	73	2.88888	5.30107
25.782	Ribono-gamma-lactone [b]	C5H8O5	1629.0	-	O,O,O-TMS	73	-	2.21266
26.523	Ribose	C5H10O5	1661.4	-	MO, O,O,O, O-TMS	73	18.56984	17.65398
28.077	Putrescine [b]	C4H12N2	1731.0	-	N,N,N, N-TMS	174	-	16.81308
28.466	2-Desoxy-pentos-3-ulose	C5H8O4	1749.1	-	2MO, O,O-TMS	73	4.64678	2.57027
28.993	3-Deoxy-arabino-hexonic acid 1,4-lactone	C6H10O5	1773.3	-	O,O,O-TMS	73	1.24872	1.52230
29.078	Ribonic acid	C5H10O6	1777.2	1799	O,O,O, O,O-TMS	73	3.23952	2.62606
29.751	3-Desoxy-pentitol [a]	C5H12O4	1808.7	-	O,O,O, O-TMS	73	2.71802	-
30.153	Galacto-hexodialdose [a]	C6H12O6	1828.3	-	2MO, O,O,O, O-TMS	160	2.76357	-
30.454	Galactofuranose [a]	C6H12O6	1842.9	1852	O,O,O, O,O-TMS	73	14.93762	-
30.993	Fructose [a]	C6H12O6	1869.1	-	MO, O,O,O, O,O-TMS	103	55.76917	-
31.214	Gluconolactone [b]	C6H10O6	1879.8	1916	O,O,O, O-TMS	73	-	10.25591
31.225	Fructose [a]	C6H12O6	1880.3	-	MO, O,O,O, O,O-TMS	103	49.85069	-
31.407	Glucose [a]	C6H12O6	1889.1	-	O,O,O, O,O-TMS	204	129.38653	-
31.422	Glucono-gamma-lactone [b]	C6H10O6	1889.8	-	O,O,O, O-TMS	73	-	6.50606
31.688	Glucose [a]	C6H12O6	1902.9	-	MO, O,O,O, O,O-TMS	319	169.75758	-
31.966	Glucose [a]	C6H12O6	1917.1	-	MO, O,O,O,	73	144.25118	-

					O,O-TMS			
33.217	Galactopyranose [a]	C6H12O6	1980.8	-	O,O,O,O,O-TMS	204	119.51640	-
33.456	Gluconic acid [b]	C6H12O7	1993.0	1997	O,O,O,O,O,O-TMS	73	-	35.72410
34.431	Hexadecanoic acid	C16H32O2	2044.7	2047	O-TMS	117	0.62709	0.34125

Note: Superscript a means only *B. cereus* secretion, superscript b means only *P. fluorescens* secretion, and no superscript means both secretions. MO was methoximation, TMS was trimethylsilyl, O was oxygen, and N was nitrogen. RI reference was derived from NIST05. Peak area data include the mean of six replicates.

Text S1. The 16S rDNA gene sequences of *Bacillus cereus* qh-35.

GGGGAGTGGCGGCAGCTATACATGCAGTCGAGCGAATGGATTAAGAGCTTGCTCTTAT
GAAGTTAGCGGCGGACGGGTGAGTAACACGTGGGTAACCTGCCCATAAGACTGGGAT
AACTCCGGGAAACCGGGGCTAATACCGGATAACATTTTGAACTGCATGGTTCGAAATT
GAAAGGCGGCTTCGGCTGTCACTTATGGATGGACCCGCGTCGCATTAGCTAGTTGGTG
AGGTAACGGCTCACCAAGGCAACGATGCGTAGCCGACCTGAGAGGGTGATCGGCCAC
ACTGGGACTGAGACACGGCCCAGACTCCTACGGGAGGCAGCAGTAGGGAATCTTCCG
CAATGGACGAAAGTCTGACGGAGCAACGCCGCGTGAGTGATGAAGGCTTTCGGGTCG
TAAAACTCTGTTGTTAGGGAAGAACAAGTGCTAGTTGAATAAGCTGGCACTGACGGTA
CCTAACCAGAAAGCCACGGCTAACTACGTGCCAGCAGCCGCGGTAATACGTAGGTGG
CAAGCGTTATCCGGAATTATTGGGCGTAAAGCGCGCGCAGGTGGTTTCTTAAGTCTGAT
GTGAAAGCCCACGGCTCAACCGTGGAGGGTCATTGGAAACTGGGAGACTTGAGTGCA
GAAGAGGAAAGTGGAATTCCATGTGTAGCGGTGAAATGCGTAGAGATATGGAGGAAC
ACCAGTGGCGAAGGCGACTTTCTGGTCTGTAACTGACACTGAGGCGCGAAAGCGTGG
GGAGCAAACAGGATTAGATACCCTGGTAGTCCACGCCGTAAACGATGAGTGCTAAGT
GTTAGAGGGTTTCCGCCCTTTAGTGCTGAAGTTAACGCATTAAGCACTCCGCCTGGGG
AGTACGGCCGCAAGGCTGAAACTCAAAGGAATTGACGGGGGCCCGCACAAGCGGTG
GAGCATGTGGTTTAATTCGAAGCAACGCGAAGAACCTTACCAGGTCTTGACATCCTCT
GAAAACCCTAGAGATAGGGCTTCTCCTTCGGGAGCAGAGTGACAGGTGGTGCATGGT
TGTCGTCAGCTCGTGTCGTGAGATGTTGGGTTAAGTCCCGCAACGAGCGCAACCCTTG
ATCTTAGTTGCCATCATTAAGTTGGGCACTCTAAGGTGACTGCCGGTGACAAACCGGA
GGAAGGTGGGGATGACGTCAAATCATCATGCCCCTTATGACCTGGGCTACACACGTGC
TACAATGGACGGTACAAAGAGCTGCAAGACCGCGAGGTGGAGCTAATCTCATAAAAC
CGTTCTCAGTTCGGATTGTAGGCTGCAACTCGCCTACATGAAGCTGGAATCGCTAGTAA
TCGCGGATCAGCATGCCGCGGTGAATACGTTCCCGGGCCTTGTACACACCGCCCGTCA
CACCACGAGAGTTTGTAACACCCGAAGTCGGTGGGGTAACCTTTTTGGAGCCAGCCG
CCTAAGTGACAGC

Text S2. The 16S rDNA gene sequences of *Pseudomonas fluorescens* gim-3.

TGGGGGCGCTACCATGCAGTCGAGCGGTAGAGAGAAGCTTGCTTCTCTTGAGAGCGG
CGGACGGGTGAGTAATGCCTAGGAATCTGCCTGGTAGTGGGGGATAACGTCCGGAAA
CGGACGCTAATACCGCATACGTCCTACGGGAGAAAGCAGGGGACCTTAGGGCCTTGC
GCTATCAGATGAGCCTAGGTCGGATTAGCTAGTTGGTGAGGTAATGGCTCACCAAGGC
GACGATCCGTAACTGGTCTGAGAGGATGATCAGTCACACTGGAACTGAGACACGGTC
CAGACTCCTACGGGAGGCAGCAGTGGGGAATATTGGACAATGGGCGAAAGCCTGATC
CAGCCATGCCGCGTGTGTGAAGAAGGTCTTCGGATTGTAAAGCACTTTAAGTTGGGAG
GAAGGGTACTTACCTAATACGTGAGTATTTTGACGTTACCGACAGAATAAGCACCGGC
TAACTCTGTGCCAGCAGCCGCGGTAATACAGAGGGTGCAAGCGTTAATCGGAATTAC
TGGGCGTAAAGCGCGCGTAGGTGGTTCGTTAAGTTGGATGTGAAATCCCCGGGCTCAA
CCTGGGAACTGCATCCAAAACTGGCGAGCTAGAGTATGGTAGAGGGTGGTGGAATTT
CCTGTGTAGCGGTGAAATGCGTAGATATAGGAAGGAACACCAGTGGCGAAGGCGACC
ACCTGGACTGATACTGACACTGAGGTGCGAAAGCGTGGGGAGCAAACAGGATTAGAT
ACCCTGGTAGTCCACGCCGTAAACGATGTCAACTAGCCGTTGGGAGCCTTGAGCTCTT
AGTGGCGCAGCTAACGCATTAAGTTGACCGCCTGGGGAGTACGGCCGCAAGGTTAAA
ACTCAAATGAATTGACGGGGGCCCGCACAAGCGGTGGAGCATGTGGTTTAATTCGAA
GCAACGCGAAGAACCTTACCAGGCCTTGACATCCAATGAACTTTCCAGAGATGGATT
GGTGCCTTCGGGAGCATTGAGACAGGTGCTGCATGGCTGTCGTCAGCTCGTGTCGTGA
GATGTTGGGTTAAGTCCCGTAACGAGCGCAACCCTTGTCCTTAGTTACCAGCACGTTAT
GGTGGGCACTCTAAGGAGACTGCCGGTGACAAACCGGAGGAAGGTGGGGATGACGT
CAAGTCATCATGGCCCTTACGGCCTGGGCTACACACGTGCTACAATGGTCGGTACAGA
GGGTTGCCAAGCCGCGAGGTGGAGCTAATCCCATAAAACCGATCGTAGTCCGGATCG
CAGTCTGCAACTCGACTGCGTGAAGTCGGAATCGCTAGTAATCGCGAATCAGAATGTC
GCGGTGAATACGTTCCCGGGCCTTGTACACACCGCCCGTCACACCATGGGAGTGGGTT
GCACCAGAAGTAGCTAGTCTAACCTTCGGGAGGACGGTACCAGCGGTGATTACGG
